# Estimation and Error Analysis for Optomechanical Inertial Sensors

**DOI:** 10.3390/s21186101

**Published:** 2021-09-11

**Authors:** Patrick Kelly, Manoranjan Majji, Felipe Guzmán

**Affiliations:** Department of Aerospace Engineering, Texas A&M University, 3141 TAMU, College Station, TX 77843-3141, USA; kelly107@tamu.edu (P.K.); mmajji@tamu.edu (M.M.)

**Keywords:** optomechanical sensors, accelerometer, estimation, error analysis, sensor design

## Abstract

A sensor model and methodology to estimate the forcing accelerations measured using a novel optomechanical inertial sensor with the inclusion of stochastic bias and measurement noise processes is presented. A Kalman filter for the estimation of instantaneous sensor bias is developed; the outputs from this calibration step are then employed in two different approaches for the estimation of external accelerations applied to the sensor. The performance of the system is demonstrated using simulated measurements and representative values corresponding to a bench-tested 3.76 Hz oscillator. It is shown that the developed methods produce accurate estimates of the bias over a short calibration step. This information enables precise estimates of acceleration over an extended operation period. These results establish the feasibility of reliably precise acceleration estimates using the presented methods in conjunction with state of the art optomechanical sensing technology.

## 1. Introduction

Accelerometers are used in countless applications including: satellite, aircraft, robotic, and automotive inertial navigation. Particularly, in the realm of satellite design these devices are used both as navigation equipment [1,2,3,4] and as scientific instrumentation [5,6,7,8,9,10,11]. In both cases the accuracy of the estimated acceleration directly impacts the performance of the system and its ability to meet critical mission requirements. There is presently a vast array of commercially available inertial sensors, developed using a diverse set of physical principles, providing a wide range of precision and sensitivity [12]. Notably, advances in optomechanics have facilitated the development of ultra-sensitive acceleration sensors with a comparatively small form factor [13]. The quality factor, a unitless parameter indicating the sharpness of the resonance peak of the oscillator [14], is of particular interest in quantifying the performance of these sensors. Previous studies indicate that such systems are capable of attaining an exceptionally high quality factor over a range of high and low frequencies, providing a broad dynamic range for acceleration sensing [15,16,17]. The use of highly accurate 3D manufacturing solutions has resulted in sensors with precise operating parameters that are uniquely suited to traditional estimation techniques without reliance on complex error models.

Inertial sensors do not directly observe forcing accelerations or torques, but rather the forces are estimated by assimilating the measurements of the dynamic response of a mechanical system strapped down to the system whose accelerations are to be measured. The fidelity of the sensor model directly impacts the quality of the estimate, and in turn the performance of the navigation system [18,19,20]. Sensor models can never perfectly characterize the behavior of the system and always possess some degree of uncertainty in the established parameters; for this reason these models are intrinsically stochastic. Since the mechanical system’s dynamical response to imposed forces is correlated with its own dynamics, which can be non-linear, the observed imposed accelerations are colored by the mechanical system dynamics. Stochastic processes used in the sensor modeling aim at decorrelating the sensor dynamics and associated modeling errors to estimate the imposed acceleration. Characterization of the sensor model’s response to these stochastic terms and their impact on performance is an essential component of practical sensor design. This analysis is routinely conducted for angular rate sensors by incorporating external measurements from attitude sensors into a Kalman filter to estimate the gyro biases and their associated steady-state statistics [21,22,23,24]. This analysis has been demonstrated to significantly improve performance in the area of spacecraft attitude determination. Similar procedures are required for estimation of accelerometer biases; namely some source of external information must be provided intermittently to conduct a calibration. This can be done using absolute position estimates from sources, such as GPS [25,26,27], or attitude estimates [28,29,30].

In this paper, a sensor model and estimators for use with state of the art optomechanical inertial sensors [16,17] are presented. Firstly, a method for sensor calibration using a generalized input acceleration is presented using a discrete time Kalman filter to estimate the instantaneous sensor bias. The information from this procedure is then fed forward to the operational phase where estimates of external accelerations are obtained. Two estimation approaches are presented for the task of estimating acceleration, each of which is compatible with the information obtained in the calibration step. These two phases constitute the operational flow of the sensor’s estimation program. Results are presented for each of these filters using representative oscillator parameters and simulated measurements in the presence of a sinusoidal external acceleration. It is shown that the calibration filter is able to provide accurate estimates of the instantaneous bias in the presence of an imperfect generalized force over a short window of time. Additionally, the operational filter (for estimates of acceleration) provides precise estimates of external acceleration whose errors follow the bias over extended periods of time. The bias contribution to the acceleration estimate error is well expressed by the associated estimate error variances.

## 2. Mathematical Formulation and Estimation Methods

### 2.1. Sensor Model

The sensor is composed of two monolithic fused silica resonators, each of which are made up of a dual-flexure supported proof mass. The dynamics governing the motion of the proof mass undergoing small deflections can be modeled as a perturbed linear harmonic oscillator. The effects of neglected vibrational modes, as well as thermomechanical and optical noise sources are then captured by a Gaussian white noise process and a bias term. The resultant equation of motion is given as:

(1) $$\ddot{x}+2\omega \zeta \dot{x}+\omega^{2}x=b\left(t\right)+n_{v}\left(t\right)+g\left(t\right)$$

where *x* is the proof mass’s displacement from the equilibrium position, g(t) is the forcing acceleration, ω is the oscillators natural frequency, and ζ is the damping ratio, which is inversely proportional to the quality factor (Q=1/2ζ). The bias term b(t) is modeled as a Wiener process:

(2) $$\dot{b}\left(t\right)=n_{u}\left(t\right)$$

where $\left\{n_{v}\left(t\right),n_{u}\left(t\right)\right\}$ are uncorrelated, zero-mean Gaussian random processes with autocorrelation functions:

(3) $$\begin{array}{cc} E[n_{v}\left(t_{i}\right)n_{v}\left(t_{j}\right)] & =\sigma_{v}^{2}\delta \left(t_{i}-t_{j}\right)\\ E[n_{u}\left(t_{i}\right)n_{u}\left(t_{j}\right)] & =\sigma_{u}^{2}\delta \left(t_{i}-t_{j}\right) \end{array}$$

where δ(t) is the Dirac delta function. The contributing process noise sources, including: thermomechanical losses and cavity drift have been investigated in references [16,17], respectively. Highly precise manufacturing processes coupled with pressure and temperature control allow for these noise sources to be modeled as Gaussian. The effect of radiation pressure from the optical measurement device is also conservatively introduced to the system using the Wiener process model for the bias. It is worth noting that strictly speaking the contribution of radiation pressure is not Gaussian, however it is commonly linearized for reduced order modeling [31,32]. For this single degree of freedom model the effect of off-axis accelerations are not considered, but could be augmented to a three-axis accelerometer configuration using this model in a straightforward manner.

The equations of motion can be expressed in state-space representation as [33]:

(4) $$\dot{X}\left(t\right)=\left[\begin{array}{ccc} 0 & 1 & 0\\ -\omega^{2} & -2\omega \zeta & 1\\ 0 & 0 & 0 \end{array}\right]X\left(t\right)+\left[\begin{array}{c} 0\\ g(t)\\ 0 \end{array}\right]+\left[\begin{array}{c} 0\\ n_{v}\left(t\right)\\ n_{u}\left(t\right) \end{array}\right].$$

In this formulation the state is defined as: $X\left(t\right)={\left[\begin{array}{ccc} x(t) & \dot{x}\left(t\right) & b(t) \end{array}\right]}^{T}$. The continuous time dynamics for the linear time invariant system can be discretized in a straightforward manner [33,34]:

(5) $$\begin{array}{cc} X_{k+1} & =\Phi \left(t_{k+1},t_{k}\right)X_{k}+\Gamma \left(t_{k+1},t_{k}\right)g_{k}+w_{k} \end{array}$$

(6) $$\begin{array}{cc} \Psi (t_{k+1},t_{k}) & =\int_{t_{k}}^{t_{k+1}}\Phi \left(t_{k+1},\tau \right)d\tau \\ \Gamma (t_{k+1},t_{k}) & ={\left[\begin{array}{ccc} \Psi_{12}\left(t_{k+1},t_{k}\right) & \Psi_{22}\left(t_{k+1},t_{k}\right) & 0 \end{array}\right]}^{T} \end{array}$$

where $X_{k}=X\left(t_{k}\right)$, and $\Phi \left(t_{k+1},t_{k}\right)=e^{A(t_{k+1}-t_{k})}$ is the state transition matrix, which is derived explicitly in Appendix A following the developments of Skelton [35]. It is assumed that g(t) is constant over a sufficiently small interval $\left(t_{k+1}-t_{k}\right)$. The discrete random process $w_{k}$ encompasses the effects of both $n_{v}\left(t\right)$ and $n_{u}\left(t\right)$, and is expressed as:

(7) $$\begin{array}{cc} w_{k} & =\int_{t_{k}}^{t_{k+1}}\Phi \left(t_{k+1},\tau \right)f\left(\tau \right)d\tau \\ f(t) & ={\left[\begin{array}{ccc} 0 & n_{v}\left(t\right) & n_{u}\left(t\right) \end{array}\right]}^{T}. \end{array}$$

The process noise can be sampled using the covariance ($Q=E[w_{k}w_{k}^{T}]$) provided in Appendix B. In conjunction with Equation (5), this allows for the state to be determined at discrete time intervals.

Observations of the oscillator are obtained using a highly sensitive laser displacement measuring interferometer which operates at a fixed sampling frequency. These measurements are expressed as a linear function of the state:

(8) $${\tilde{y}}_{k}=HX_{k}+\nu_{k}=x_{k}+\nu_{k}$$

where $H=\left[\begin{array}{ccc} 1 & 0 & 0 \end{array}\right]$ is the measurement mapping matrix. The measurement noise ($\nu_{k}$) made up of shot noise, electric readout noise, and optical frequency noise are taken to be zero-mean Gaussian with variance $\sigma_{m}^{2}$, and $\nu_{k}$ is Gaussian white noise with variance $\sigma_{m}^{2}$. It is important to note that g(t) is made up of all frequencies of interest, while the observable frequencies of the sensor are limited by the oscillator frequency, the sampling frequency, and the relevant model uncertainties. Therefore, only a component of the forcing acceleration is sensed by the accelerometer.

### 2.2. Calibration Phase: Estimating the Sensor Bias

The accelerometer bias must be monitored to prevent the accumulated build-up of errors in the estimate of acceleration. To do this a calibration step must be conducted on a reasonable basis. One such method is to carefully apply an input to the sensor over a specified time and observe the response of the system. Knowing that the acceleration cannot be perfectly delivered, we define the provided input as:

(9) $$g\left(t\right)=\bar{g}\left(t\right)+n_{g}\left(t\right)$$

where $\bar{g}\left(t\right)$ is the desired acceleration at time *t*, and $n_{g}\left(t\right)$ is a zero-mean Gaussian process with variance $\sigma_{g}^{2}$. The discrete sensor dynamics given in Equation (5) can now be augmented with the imperfect input acceleration:

(10) $$\begin{array}{cc} X_{k+1} & =\Phi \left(t_{k+1},t_{k}\right)X_{k}+\Gamma \left(t_{k+1},t_{k}\right){\bar{g}}_{k}+w_{k} \end{array}$$

where $w_{k}$ is defined in the same way as Equation (7) with the addition of $n_{g}\left(t\right)$:

(11) $$f\left(t\right)={\left[\begin{array}{ccc} 0 & n_{g}\left(t\right)+n_{v}\left(t\right) & n_{u}\left(t\right) \end{array}\right]}^{T}.$$

Because the input noise $\left(n_{g}\left(t\right)\right)$ and the process noise terms $\left\{n_{v}\left(t\right),n_{u}\left(t\right)\right\}$ are independent, the Q matrix derived in Appendix B would be of the same form, with the sum of $\sigma_{g}^{2}$ and $\sigma_{v}^{2}$ taking the place of $\sigma_{v}^{2}$. With the dynamics in this form, the discrete-time Kalman filter can be used to sequentially estimate the state using displacement measurements defined in Equation (8) [34,36,37]. The update step, which is conducted when a new measurement is incorporated is given as:

(12) $$\begin{array}{cc} {\hat{X}}_{k}^{+} & ={\hat{X}}_{k}^{-}+K_{k}\left({\tilde{y}}_{k}-H\;{\hat{X}}_{k}^{-}\right)\\ P_{k}^{+} & =\left(I-K_{k}H\right)P_{k}^{-}.\\ K_{k} & =P_{k}^{-}H^{T}{\left[HP_{k}^{-}H^{T}+R\right]}^{-1} \end{array}$$

where the caret corresponds to the estimate of the indicated quantity and P is the estimate error covariance matrix. After a measurement is incorporated it is propagated over the sampling interval $\left(t_{k+1}-t_{k}\right)$. The propagation equations are given as:

(13) $$\begin{array}{cc} {\hat{X}}_{k+1}^{-} & =\Phi \left(t_{k+1},t_{k}\right){\hat{X}}_{k}^{+}+\Gamma \left(t_{k+1},t_{k}\right){\bar{g}}_{k}\\ P_{k+1}^{-} & =\Phi \left(t_{k+1},t_{k}\right)P_{k}^{+}\Phi^{T}\left(t_{k+1},t_{k}\right)+Q. \end{array}$$

Using this method, an estimate of the sensor bias and the variance of the bias estimate error can be obtained sequentially in the calibration step. Provided a reasonably steady input of an admissible magnitude is applied, the filter will converge to steady-state behavior quite rapidly. With this in mind, the convergent value of P can be found by solving the discrete Riccati equation [38,39,40]:

(14) $$P=\Phi \left(t_{k+1},t_{k}\right)\left[I-KH\right]P\Phi^{T}\left(t_{k+1},t_{k}\right)+Q.$$

In doing so, a closed-form solution for the expected filter performance can be obtained based on oscillator parameters, sampling frequency, and process noise parameters. This steady-state analysis is important for sensor design and component selection. Accuracies of the displacement-sensing interferometers, process models of the mechanical elements, and the signal processing specifications can be designed using the mathematics and methods of the steady-state analysis.

### 2.3. Operation Phase: Estimating the Forcing Acceleration

The results obtained from the calibration step can now be incorporated to develop procedure for the estimation of the forcing acceleration using a series of displacement measurements defined in Equation (8). Upon completion of the calibration step, it is assumed that the bias estimate ($\hat{b}$) is itself unbiased. Under this assertion, the sensor bias is given as:

(15) $$\begin{array}{cc} b(t_{0}) & =\hat{b}+n_{b0}\\ b(t_{k}) & =b\left(t_{0}\right)+\int_{t_{0}}^{t_{k}}n_{u}\left(\tau \right)d\tau \end{array}$$

where $t_{0}$ is the time of calibration and $n_{b0}$ is a zero-mean Gaussian random variable with variance $\sigma_{b0}^{2}$. Between calibrations the bias estimate is not updated, so $\hat{b}$ does not change. Because $n_{b0}$ and $n_{u}\left(t\right)$ are both zero mean, the error in the bias estimate between calibrations will remain zero mean; however the variance in the estimate will grow [41]. The statistical quantities governing this growth are given by:

(16) $$\begin{array}{cc} \; & E[b_{k}-\hat{b}]=0\\ \; & E\left[\left(b_{k}-\hat{b}\right){\left(b_{k}-\hat{b}\right)}^{T}\right]=\sigma_{b0}^{2}+\left(t_{k}-t_{0}\right)\sigma_{u}^{2}. \end{array}$$

This growth in bias uncertainty between calibrations will be shown to degrade the accuracy of the subsequent acceleration estimate as the time since calibration increases. Using this instantaneous definition for the bias term, the discrete dynamics in Equation (5) can be rewritten as:

(17) $$\begin{array}{cc} \left[\begin{array}{c} x_{k+1}\\ {\dot{x}}_{k+1} \end{array}\right] & =\left[\begin{array}{cc} \Phi_{11} & \Phi_{12}\\ \Phi_{21} & \Phi_{22} \end{array}\right]\left[\begin{array}{c} x_{k}\\ {\dot{x}}_{k} \end{array}\right]+\left[\begin{array}{c} \Psi_{12}\\ \Psi_{22} \end{array}\right]g_{k}+\left[\begin{array}{c} \Phi_{13}\\ \Phi_{23} \end{array}\right]b_{k}+\int_{t_{k}}^{t_{k+1}}\left[\begin{array}{c} n_{v}\left(\tau \right)\Phi_{12}\left(t_{k+1},\tau \right)\\ n_{v}\left(\tau \right)\Phi_{22}\left(t_{k+1},\tau \right) \end{array}\right]d\tau . \end{array}$$

Using this reduced form of the discrete dynamics, two estimators will now be presented.

#### 2.3.1. Least Squares Formulation

The first estimator considered is a moving least squares method using a batch of N+1 measurements obtained from the laser interferometer, which are defined in Equation (8). A measurement obtained at n∈{0,1,2,…,N|N≥2} steps ahead of $t_{k}$ is then expressed as the following:

(18) $$\begin{array}{cc} y_{k+n} & =\left[\begin{array}{ccc} \Phi_{11}^{\left(n\right)} & \Phi_{12}^{\left(n\right)} & \Psi_{12}^{\left(n\right)} \end{array}\right]\left[\begin{array}{c} x_{k}\\ {\dot{x}}_{k}\\ g_{k} \end{array}\right]+\Phi_{13}^{\left(n\right)}\hat{b}+{\tilde{\nu }}_{k+n}\\ {\tilde{\nu }}_{k+n} & =\nu_{k+n}+\Phi_{13}^{\left(n\right)}\left(b_{k}-\hat{b}\right)+\int_{t_{k}}^{t_{k+n}}\Phi_{13}\left(t_{\left(k+n\right)},\tau \right)n_{v}\left(\tau \right)d\tau , \end{array}$$

where $\Phi_{ij}^{\left(n\right)}$ and $\Psi_{ij}^{\left(n\right)}$ are used as shorthand for $\Phi_{ij}(t_{\left(k+n\right)},t_{k})$ and $\Psi_{ij}(t_{\left(k+n\right)},t_{k})$, respectively. Using this, a set of measurements can then be expressed as a function of the instantaneous oscillator state and forcing acceleration:

(19) $$\left[\begin{array}{c} y_{k}\\ y_{k+1}\\ \vdots \\ y_{k+N} \end{array}\right]=\left[\begin{array}{ccc} 1 & 0 & 0\\ \Phi_{11}^{\left(1\right)} & \Phi_{12}^{\left(1\right)} & \Psi_{12}^{\left(1\right)}\\ & \vdots & \\ \Phi_{11}^{\left(n\right)} & \Phi_{12}^{\left(n\right)} & \Psi_{12}^{\left(n\right)} \end{array}\right]\left[\begin{array}{c} x_{k}\\ {\dot{x}}_{k}\\ g_{k} \end{array}\right]+\left[\begin{array}{c} 0\\ \Phi_{13}^{\left(1\right)}\\ \vdots \\ \Phi_{13}^{\left(n\right)} \end{array}\right]\hat{b}+\left[\begin{array}{c} {\tilde{\nu }}_{k}\\ {\tilde{\nu }}_{k+1}\\ \vdots \\ {\tilde{\nu }}_{k+N} \end{array}\right].$$

Now, the minimum variance estimate of ${\left[\begin{array}{ccc} x_{k} & {\dot{x}}_{k} & g_{k} \end{array}\right]}^{T}$ can be determined from the set of measurements:

(20) $$\begin{array}{cc} \left[\begin{array}{c} {\hat{x}}_{k}\\ {\hat{\dot{x}}}_{k}\\ {\hat{g}}_{k} \end{array}\right] & ={\left[{\tilde{H}}_{k}^{T}R^{-1}{\tilde{H}}_{k}\right]}^{-1}{\tilde{H}}_{k}^{T}R^{-1}\left(\tilde{y}-\eta_{k}\hat{b}\right)\\ P & ={\left[{\tilde{H}}_{k}^{T}R^{-1}{\tilde{H}}_{k}\right]}^{-1} \end{array}$$

(21) $$\begin{array}{ccccc} \; & {\tilde{H}}_{k}=\left[\begin{array}{ccc} 1 & 0 & 0\\ \Phi_{11}^{\left(1\right)} & \Phi_{12}^{\left(1\right)} & \Psi_{12}^{\left(1\right)}\\ & \vdots & \\ \Phi_{11}^{\left(n\right)} & \Phi_{12}^{\left(n\right)} & \Psi_{12}^{\left(n\right)} \end{array}\right]\; & \; & \tilde{y}=\left[\begin{array}{c} y_{k}\\ y_{k+1}\\ \vdots \\ y_{k+N} \end{array}\right]\; & \eta_{k}=\left[\begin{array}{c} 0\\ \Phi_{13}^{\left(1\right)}\\ \vdots \\ \Phi_{13}^{\left(n\right)} \end{array}\right] \end{array}$$

where the caret corresponds to the estimate of the indicated term and R is the measurement noise covariance. The elements of R are given in indicial notation as:

(22) $$R_{ij}=\sigma_{m}^{2}\delta_{ij}+\Phi_{13}^{\left(i\right)}\Phi_{13}^{\left(j\right)}\left(\sigma_{b0}^{2}+\sigma_{u}^{2}\left(t_{k}-t_{0}\right)\right)+\sigma_{v}^{2}\int_{t_{k}}^{t_{k+i}}\Phi_{12}^{2}\left(t_{k+i},\tau \right)d\tau$$

where i≤j and $\delta_{ij}$ is the Kronecker delta. A closed form solution for the integral in this expression is derived in Appendix B. The measurement noise has an explicit dependence on time, and as a result will increase with the time since last calibration. As a result the certainty in the estimate of acceleration will intuitively degrade during operation, necessitating periodic calibration. Provided the user has some operational requirements on the acceleration estimates, the calibration cycle can be determined explicitly in this manner.

#### 2.3.2. Kalman Filter Formulation

An estimator for the determination of the oscillator state and forcing acceleration can also be developed using a Kalman filter approach. Returning to Equation (17), the dynamics can be expressed as:

(23) $$\begin{array}{cc} \left[\begin{array}{c} x_{k+1}\\ {\dot{x}}_{k+1} \end{array}\right] & =\left[\begin{array}{cc} \Phi_{11} & \Phi_{12}\\ \Phi_{21} & \Phi_{22} \end{array}\right]\left[\begin{array}{c} x_{k}\\ {\dot{x}}_{k} \end{array}\right]+\left[\begin{array}{c} \Psi_{12}\\ \Psi_{22} \end{array}\right]\left(g_{k}+\hat{b}\right)+{\tilde{w}}_{k}\\ {\tilde{w}}_{k} & =\left[\begin{array}{c} \Phi_{13}\\ \Phi_{23} \end{array}\right]\left(b_{k}-\hat{b}\right)+\int_{t_{k}}^{t_{k+1}}\left[\begin{array}{c} n_{v}\left(\tau \right)\Phi_{12}\left(t_{k+1},\tau \right)\\ n_{v}\left(\tau \right)\Phi_{22}\left(t_{k+1},\tau \right) \end{array}\right]d\tau . \end{array}$$

The new process noise covariance $\tilde{Q}$, which has been augmented with the uncertainty in the instantaneous value of the bias, is given by the following:

(24) $$\begin{array}{cc} \tilde{Q} & =\left[\begin{array}{cc} \Phi_{13}^{2} & \Phi_{13}\Phi_{23}\\ \Phi_{23}\Phi_{13} & \Phi_{23}^{2} \end{array}\right]\left(\sigma_{b0}^{2}+\sigma_{u}^{2}\left(t_{k}-t_{0}\right)\right)\\ \; & +\sigma_{v}^{2}\left[\begin{array}{cc} \int_{t_{k}}^{t_{k+1}}\Phi_{12}^{2}\left(t_{k+1},\tau \right)d\tau & \int_{t_{k}}^{t_{k+1}}\Phi_{12}\left(t_{k+1},\tau \right)\Phi_{22}\left(t_{k+1},\tau \right)d\tau \\ \int_{t_{k}}^{t_{k+1}}\Phi_{12}\left(t_{k+1},\tau \right)\Phi_{22}\left(t_{k+1},\tau \right)d\tau & \int_{t_{k}}^{t_{k+1}}\Phi_{22}^{2}\left(t_{k+1},\tau \right)d\tau \end{array}\right] \end{array}$$

where the integrals contained in the second matrix are derived in Appendix B. The discrete dynamics can then be rearranged to include the instantaneous forcing acceleration as an additional state. In the absence of a model for g(t), the estimate must be driven by the incorporation of new measurements. This can be done by tuning the process noise associated with the channel to an appropriately scaled value (α).

(25) $$\begin{array}{cc} \left[\begin{array}{c} x_{k+1}\\ {\dot{x}}_{k+1}\\ g_{k+1} \end{array}\right] & =\left[\begin{array}{ccc} \Phi_{11} & \Phi_{12} & \Psi_{12}\\ \Phi_{21} & \Phi_{22} & \Psi_{22}\\ 0 & 0 & 1 \end{array}\right]\left[\begin{array}{c} x_{k}\\ {\dot{x}}_{k}\\ g_{k} \end{array}\right]+\left[\begin{array}{c} \Psi_{12}\\ \Psi_{22}\\ 0 \end{array}\right]\hat{b}+\left[\begin{array}{c} {\tilde{w}}_{k}\\ 0 \end{array}\right] \end{array}$$

Using these discrete dynamics, the Kalman filter can be run with slight modifications to the update and propagation equations given in Equations (12) and (13), respectively.

(26) $$\begin{array}{cc} {\hat{X}}_{k}^{+} & ={\hat{X}}_{k}^{-}+K_{k}\left({\tilde{y}}_{k}-H\;{\hat{X}}_{k}^{-}\right)\\ P_{k}^{+} & =\left(I-K_{k}H\right)P_{k}^{-}.\\ K_{k} & =P_{k}^{-}H^{T}{\left[HP_{k}^{-}H^{T}+R\right]}^{-1}, \end{array}$$

(27) $$\begin{array}{cc} {\hat{X}}_{k+1}^{-} & =\Phi \left(t_{k+1},t_{k}\right){\hat{X}}_{k}^{+}+\Gamma \left(t_{k+1},t_{k}\right)\hat{b}\\ P_{k+1}^{-} & =\Phi \left(t_{k+1},t_{k}\right)P_{k}^{+}\Phi^{T}\left(t_{k+1},t_{k}\right)+\left[\begin{array}{cc} \tilde{Q} & 0\\ 0 & \alpha \end{array}\right], \end{array}$$

**Figure 1:**
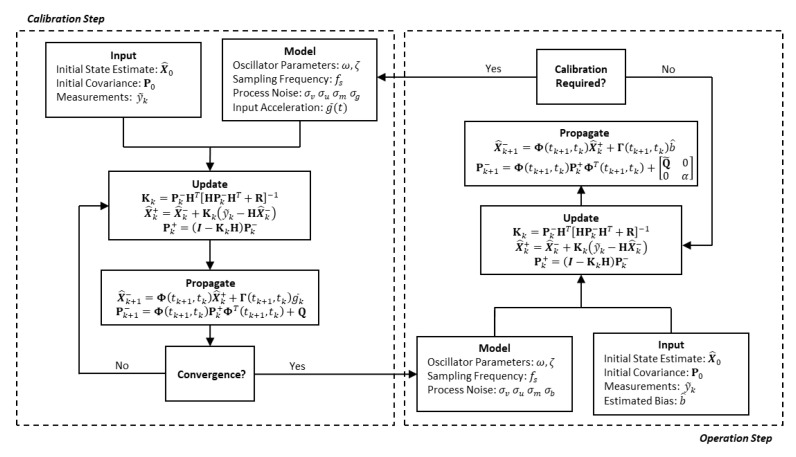
Flowchart of the calibration and operation Kalman filters. As shown, the outputs of each filter are provided as inputs to the other. Equation (20) can easily be substituted into the calibration step to use the lerast squares solution in place of the Kalman filter.

Note here that $\Phi (t_{k+1},t_{k})$ provided in Appendix A is identical to matrix in Equation (25), which allows for its use in the propagation equations above. Like the calibration step, the filter is run using displacement measurements of the proof mass given in Equation (8), which each possess measurement noise covariance $R=\sigma_{m}^{2}$. Figure 1 summarizes the developments for the calibration and operation filter and provides the framework for intended use.

## 3. Numerical Simulation Results

The combined calibration and acceleration estimation procedure is implemented and tested using a simulated dataset generated using the sensor model developed in Section 2.1. The oscillator parameters that define the sensor response are provided in Table 1. These parameters correspond to experimental results obtained from benchtesting of a prototype sensor [16,17]. The parameters that define the stochastic processes driving the system $\left\{\sigma_{v},\sigma_{u}\right\}$ are provided in Table 2. Using this information a truth model has been generated, from which the displacement time history has been extracted and corrupted by noise to create a simulated measurement set. These noisy measurements are then provided to the calibration and acceleration estimation programs.

**Figure 2:**
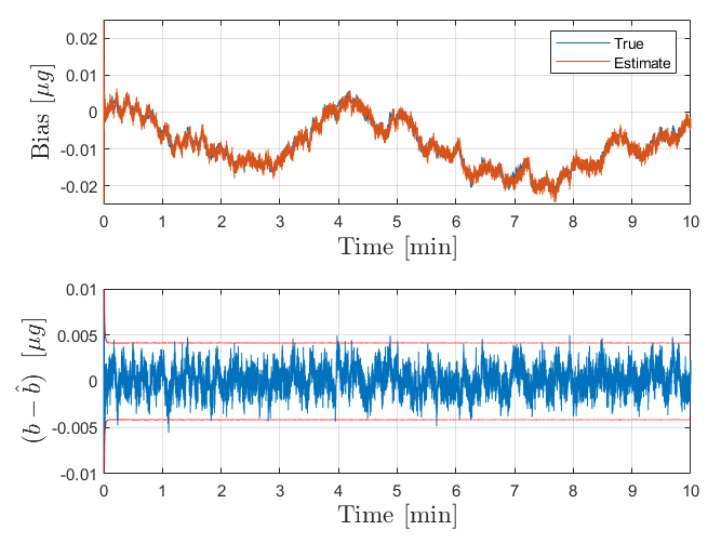
Results for the bias estimate obtained from the simulated calibration phase. The top figure shows the true and estimated bias over the calibration time interval of 10 min. On the bottom, the error in the bias estimate is plotted in blue with the corresponding 3σ bounds in red.

Firstly, the calibration step was conducted over a span of 10 min using a constant input acceleration of $\bar{g}=1\times 10^{-6}$ m/s${}^{2}$. The input noise density corresponds to 10% error in delivered acceleration, and is provided in Table 2. The initial estimate of the bias is significantly offset from the true value (1 m/s${}^{2}$). The true and estimated bias during the calibration are plotted with the bias estimate error and corresponding 3σ bounds in Figure 2. The filter is able to converge in the span of a few seconds, producing an unbiased estimate with root mean square error of 1.9816 ng. The converged variance of the bias estimate error (provided in Table 2) is then fed forward to the acceleration estimation procedure in the next step.

**Figure 3:**
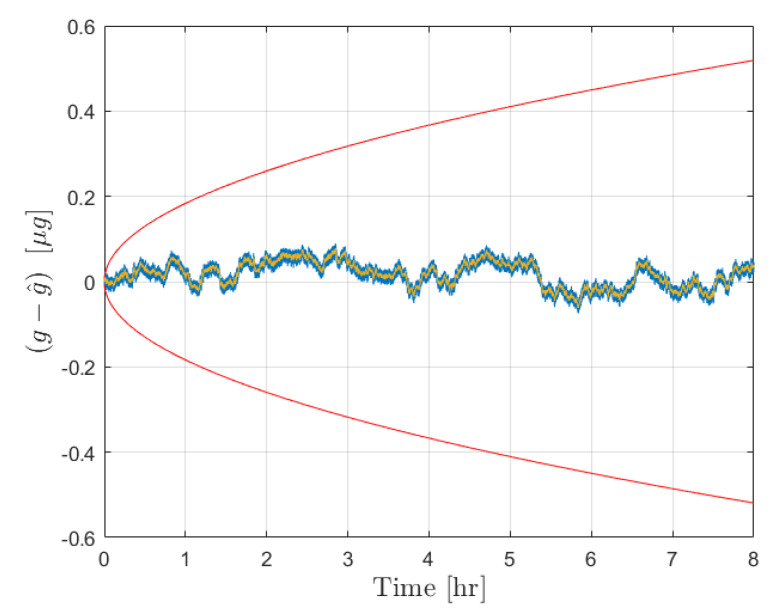
Results for the forcing acceleration estimate error from the moving least squares procedure. Estimate error is plotted in blue with the associated 3σ bounds in red. This is accompanied by the true bias time history in yellow.

**Figure 4:**
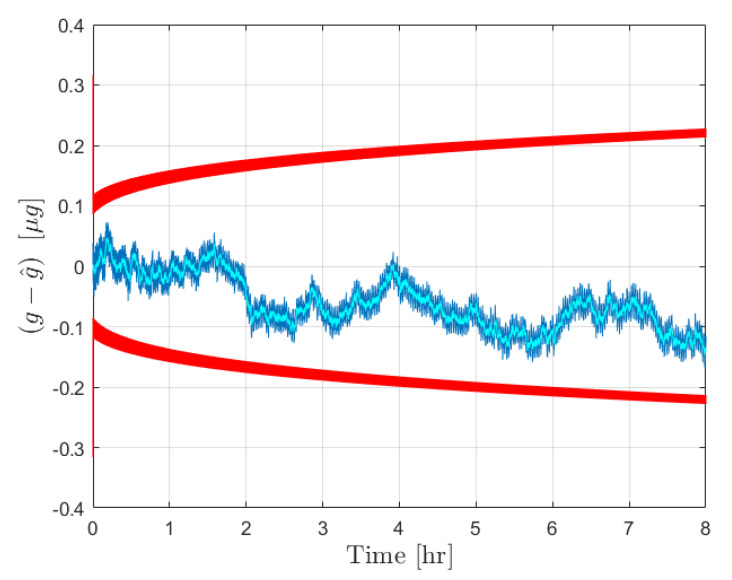
Results for the forcing acceleration estimate error from the Kalman filter. Estimate error is plotted in blue with the associated 3σ bounds in red. This is accompanied by the true bias time history in cyan.

After the calibration phase is implemented, the acceleration estimation step is carried out over an extended operation interval of 8 h. The initial true bias was set to zero, while $\hat{b}$ was set as a random drawing from $N(0,\sigma_{b0}^{2})$. The external acceleration was defined as a sinusoid of amplitude $1\times 10^{-5}$ m/s${}^{2}$ and frequency 1/100 Hz. The results for the error in estimated acceleration and the 3σ bounds are provided in Figure 3 for the moving least squares procedure and in Figure 4 for the Kalman filter. The estimate errors for both filters start as zero mean; however, as time increases the influence of the bias on the estimate error becomes apparent in both cases. This is expected, as the filter is initialized with a constant $\hat{b}$ for the initial time, and beyond that point the uncertainty in b(t) is reflected by the growth in the covariance. In both cases the estimate error is contained within the plotted 3σ bounds. The acceleration estimates can be deemed reliable until the 3σ bounds become sufficiently large to necessitate calibration, at which point the new bias estimate and $\sigma_{b0}$ can be fed forward to continue operation.

To quantify the filter performance in a manner that is independent of the specific realization of the bias random process, the procedure has been conducted for both estimation approaches in a series of 10,000, 1 h trials to generate statistical measures of performance. The results from this Monte Carlo analysis are presented in Table 3.

## 4. Discussion

The sensor model developed in Section 2 has been employed to simulate noisy sensor dynamics and measurements. Using these measurements, both the calibration and operation phase estimation procedures have been conducted in series. Figure 2 shows the results obtained in the calibration phase. It is shown that the filter is able to converge to steady-state operation in less than 5 s, in spite of poor a priori knowledge of the bias state. It is shown that the estimate tracks the true value well over the extended 10 min operating window.

**Figure 5:**
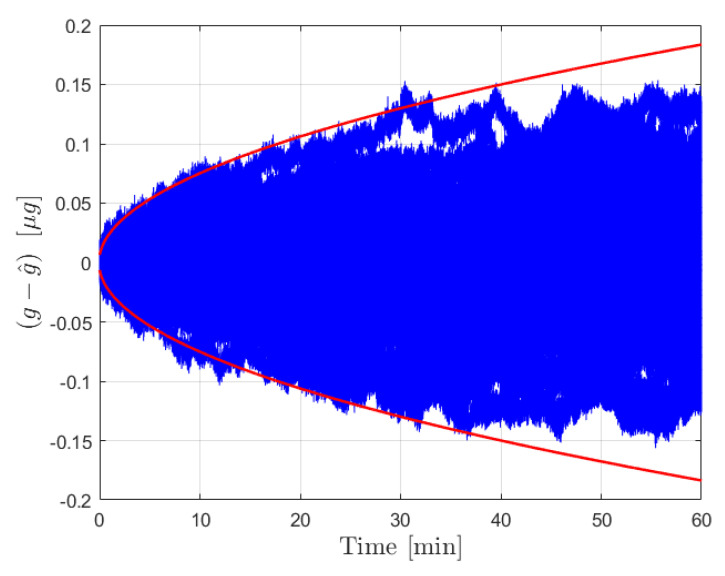
Results for the forcing acceleration estimate error from the moving least squares procedure for 100 Monte Carlo iterations. Estimate error is plotted in blue with the associated 3σ bounds in red.

**Figure 6:**
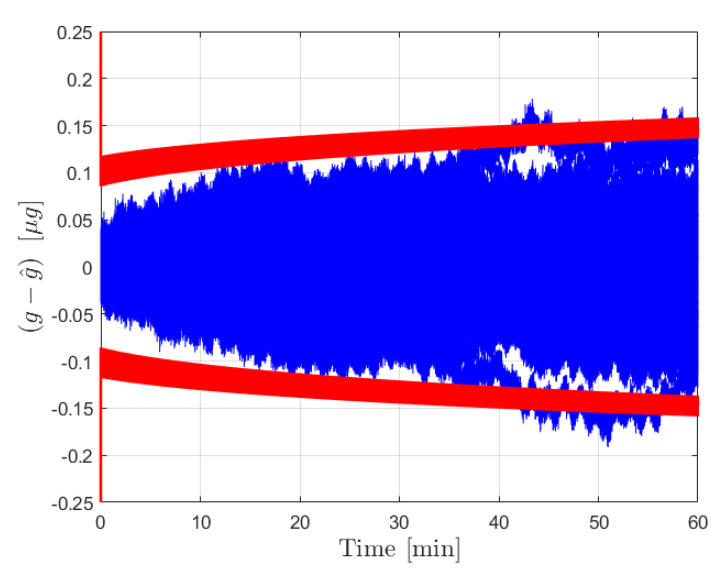
Results for the forcing acceleration estimate error from the Kalman filter procedure for 100 Monte Carlo iterations. Estimate error is plotted in blue with the associated 3σ bounds in red.

Figure 3 shows the results from the operation stage using the moving least squares formulation over a period of 8 h without calibration. During this time, the covariance grows significantly as the certainty in the bias estimate provided by the calibration is diminished. The resultant estimate error is shown to be dominated by the effect of the bias, which is plotted in yellow. This same procedure was conducted over a 1 h window for 10,000 Monte Carlo iterations, 100 of which are plotted with the 3σ bounds in Figure 5. The figure shows that the covariance is consistent with the expected results in that the errors are bounded by red curves. An important observation is that the user can determine the required calibration cycle based on required level of accuracy of the acceleration estimates. Figure 4 shows the results from the operation stage using the Kalman filter formulation. The results here mirror those of the least squares formulation. The estimate error is primarily driven by the bias term over the 8 h period, and the estimate error is contained within the 3σ bounds which are plotted in red. The main distinction between the two is the tuning of the parameter α for the process noise covariance. Figure 6 shows the results from 100 Monte Carlo iterations using the Kalman filter. As is shown, the covariance scaling is consistent with the acceleration estimate error results obtained.

## 5. Conclusions

Inertial navigation is a crucial component to the future of autonomy across several disciplines. It is required that accurate and precise measurements of acceleration and angular rates be acquired in spite of various sources of error to conduct this procedure effectively. In this paper a stochastic model for novel optomechanical inertial sensors is presented and utilized to derive procedures for the estimation of sensor bias during a calibration step, and forcing accelerations during operation. These procedures are developed to be fully compatible, as outputs from the calibration filter are used directly in the operation phase, thus constituting a full sequence for accurate acceleration measurement. This procedure is demonstrated using representative sensor parameters and simulated process and measurement noise. The simulation results verify that the Kalman filter used for calibration is able to produce a highly accurate estimate of the instantaneous bias in the presence of an imperfect input, and that both operational filters produce precise estimates of acceleration. The acceleration estimation errors induced by the bias as it evolves over the operational window are shown to be fully captured by the covariance; this result is proposed as a quantitative method to determine the required calibration cycle to maintain a desired degree of accuracy. In summary, this work addresses the critical concerns of: how to estimate the forcing acceleration in the presence of a stochastic bias term, how to estimate this bias term during a calibration phase, and how regularly must calibration phases be conducted to retain the desired degree of precision.

## Figures and Tables

**Table 1:** Oscillator parameters for optomechanical inertial sensor.

Term	Value	Units
ω	3.76	Hz
*Q*	$1.14\times 10^{5}$	
$f_{s}$	30.5	Hz

**Table 2:** Random process parameters.

Term	Value	Units
$\sigma_{v}$	$1\times 10^{-9}$	m/s $\sqrt{\mathrm{Hz}}$
$\sigma_{u}$	$1\times 10^{-8}$	m/s${}^{2}\sqrt{\mathrm{Hz}}$
$\sigma_{m}$	$1\times 10^{-11}$	m
$\sigma_{g}$	$1.8107\times 10^{-8}$	m/s $\sqrt{\mathrm{Hz}}$
$\sigma_{b0}$	$1.3506\times 10^{-8}$	m/s${}^{2}$

**Table 3:** Key performance statistics from 10,000 Monte Carlo trials of both acceleration estimation procedures.

Term	Value	Units
Least Squares Error Mean	$7.7990\times 10^{-5}$	μg
Least Squares Error Std Dev	$3.6446\times 10^{-2}$	μg
Kalman Filter Error Mean	$3.7493\times 10^{-5}$	μg
Kalman Filter Error Std Dev	$3.5476\times 10^{-2}$	μg

## Appendix A. State Transition Matrix

The state transition matrix $\left(\Phi (t,t_{0})\right)$ provides the fundamental solution to a homogeneous linear state-space system, mapping the state at some reference time ($t_{0}$) into the solution space at a future time (t). For a state-space defined as:

(A1) $$\dot{x}\left(t\right)=Ax\left(t\right)+Bu\left(t\right)$$

where {A,B} are constant matrices, the state transition matrix is determined by the matrix exponential:

(A2) $$\Phi \left(t,t_{0}\right)=e^{A(t-t_{0})}.$$

The resultant solution flow of the non-homogeneous system given in Equation (A1) is then determined with the STM:

(A3) $$x\left(t\right)=\Phi \left(t,t_{0}\right)x\left(t_{0}\right)+B\int_{t_{0}}^{t}\Phi \left(t,\tau \right)u\left(\tau \right)d\tau$$

as is seen in the formulation of Equation (5). For the continuous LTI system defined in Equation (4) the matrix exponential provides the following analytic STM:

(A4) $$\Phi =\left[\begin{array}{ccc} \Phi_{11} & \Phi_{12} & \Phi_{13}\\ \Phi_{21} & \Phi_{22} & \Phi_{23}\\ 0 & 0 & 1 \end{array}\right]$$

(A5) $$p_{1}=\omega \sqrt{1-\zeta^{2}}\;\;\;\;\;\;p_{2}=\omega \zeta$$

(A6) $$\begin{array}{cc} \Phi_{11} & =\frac{1}{p_{1}}e^{-p_{2}\left(t-t_{0}\right)}\left[p_{1}\cos\left(p_{1}\left(t-t_{0}\right)\right)+p_{2}\sin\left(p_{1}\left(t-t_{0}\right)\right)\right]\\ \Phi_{12} & =\frac{1}{p_{1}}e^{-p_{2}\left(t-t_{0}\right)}\sin\left(p_{1}\left(t-t_{0}\right)\right)\\ \Phi_{13} & =\frac{1}{\omega^{2}}\left[1-\frac{1}{p_{1}}e^{-p_{2}\left(t-t_{0}\right)}\left(p_{1}\cos\left(p_{1}\left(t-t_{0}\right)\right)+p_{2}\sin\left(p_{1}\left(t-t_{0}\right)\right)\right)\right] \end{array}$$

(A7) $$\begin{array}{cc} \Phi_{21} & =-\frac{\omega^{2}}{p_{1}}e^{-p_{2}\left(t-t_{0}\right)}\sin\left(p_{1}\left(t-t_{0}\right)\right)\\ \Phi_{22} & =\frac{1}{p_{1}}e^{-p_{2}\left(t-t_{0}\right)}\left[p_{1}\cos\left(p_{1}\left(t-t_{0}\right)\right)-p_{2}\sin\left(p_{1}\left(t-t_{0}\right)\right)\right]\\ \Phi_{23} & =\frac{1}{p_{1}}e^{-p_{2}\left(t-t_{0}\right)}\sin\left(p_{1}\left(t-t_{0}\right)\right). \end{array}$$

Additionally, to account for the effect of the forcing function, the integral of $\Phi (t,t_{0})$ is required, particularly the elements that make up $\Gamma (t,t_{0})$ which account for the effect of the forcing acceleration and bias terms.

(A8) $$\Psi \left(t,t_{0}\right)=\int_{t_{0}}^{t}\Phi \left(t,\tau \right)d\tau$$

(A9) $$\begin{array}{cc} \Psi_{11} & =\frac{1}{p_{1}\omega^{2}}\left[2p_{1}p_{2}+e^{-p_{2}dt}\left(\left(p_{1}^{2}-p_{2}^{2}\right)\sin\left(p_{1}\left(t-t_{0}\right)\right)-2p_{1}p_{2}\cos\left(p_{1}\left(t-t_{0}\right)\right)\right)\right]\\ \Psi_{12} & =\frac{1}{\omega^{2}}\left[1-\frac{1}{p_{1}}e^{-p_{2}\left(t-t_{0}\right)}\left(p_{1}\cos\left(p_{1}\left(t-t_{0}\right)\right)+p_{2}\sin\left(p_{1}\left(t-t_{0}\right)\right)\right)\right]\\ \Psi_{13} & =\frac{1}{\omega^{2}}-\frac{1}{p_{1}\omega^{4}}\left[2p_{1}p_{2}+e^{-p_{2}dt}\left(\left(p_{1}^{2}-p_{2}^{2}\right)\sin\left(p_{1}\left(t-t_{0}\right)\right)-2p_{1}p_{2}\cos\left(p_{1}\left(t-t_{0}\right)\right)\right)\right]\\ \Psi_{21} & =-\left[1-\frac{1}{p_{1}}e^{-p_{2}\left(t-t_{0}\right)}\left(p_{1}\cos\left(p_{1}\left(t-t_{0}\right)\right)+p_{2}\sin\left(p_{1}\left(t-t_{0}\right)\right)\right)\right]\\ \Psi_{22} & =\frac{1}{p_{2}}e^{-p_{2}\left(t-t_{0}\right)}\sin\left(p_{1}\left(t-t_{0}\right)\right)\\ \Psi_{23} & =\frac{1}{\omega^{2}}\left[1-\frac{1}{p_{1}}e^{-p_{2}\left(t-t_{0}\right)}\left(p_{1}\cos\left(p_{1}\left(t-t_{0}\right)\right)+p_{2}\sin\left(p_{1}\left(t-t_{0}\right)\right)\right)\right]\\ \Psi_{31} & =0\\ \Psi_{32} & =0\\ \Psi_{33} & =(t-t_{0}) \end{array}$$

## Appendix B. Process Noise Covariance

The process noise vector $w_{k}$ defined in Equation (5) is composed of a Gaussian white noise process, and a random walk. The instantaneous value of this term is given by:

(A10) $$\begin{array}{cc} w_{k} & =\int_{t_{k}}^{t_{k+1}}\left[\begin{array}{ccc} \Phi_{11}\left(t_{k+1},\tau \right) & \Phi_{12}\left(t_{k+1},\tau \right) & \Phi_{13}\left(t_{k+1},\tau \right)\\ \Phi_{21}\left(t_{k+1},\tau \right) & \Phi_{22}\left(t_{k+1},\tau \right) & \Phi_{23}\left(t_{k+1},\tau \right)\\ \Phi_{31}\left(t_{k+1},\tau \right) & \Phi_{32}\left(t_{k+1},\tau \right) & \Phi_{33}\left(t_{k+1},\tau \right) \end{array}\right]\left[\begin{array}{c} 0\\ n_{v}\left(\tau \right)\\ n_{u}\left(\tau \right) \end{array}\right]d\tau \\ \; & =\int_{t_{k}}^{t_{k+1}}\left[\begin{array}{c} \Phi_{12}\left(t_{k+1},\tau \right)n_{v}\left(\tau \right)+\Phi_{13}\left(t_{k+1},\tau \right)n_{u}\left(\tau \right)\\ \Phi_{22}\left(t_{k+1},\tau \right)n_{v}\left(\tau \right)+\Phi_{23}\left(t_{k+1},\tau \right)n_{u}\left(\tau \right)\\ n_{u}\left(\tau \right) \end{array}\right]d\tau \end{array}$$

For any admissible value of *k*, $w_{k}$ is a zero-mean Gaussian random vector with covariance $Q=E[w_{k}w_{k}^{T}]$. As such, the elements of this covariance matrix are required to define the multivariate distribution from which drawings of $w_{k}$ are taken. By applying the definitions in Equation (3), the elements of Q are given as:

(A11) $$\begin{array}{cc} Q_{11} & =\sigma_{v}^{2}\int_{t_{k}}^{t_{k+1}}\Phi_{12}^{2}\left(t_{k+1},\tau \right)d\tau +\sigma_{u}^{2}\int_{t_{k}}^{t_{k+1}}\Phi_{13}^{2}\left(t_{k+1},\tau \right)d\tau \\ Q_{12} & =Q_{21}=\sigma_{v}^{2}\int_{t_{k}}^{t_{k+1}}\Phi_{12}\left(t_{k+1},\tau \right)\Phi_{22}\left(t_{k+1},\tau \right)d\tau +\sigma_{u}^{2}\int_{t_{k}}^{t_{k+1}}\Phi_{13}\left(t_{k+1},\tau \right)\Phi_{23}\left(t_{k+1},\tau \right)d\tau \\ Q_{13} & =Q_{31}=\sigma_{u}^{2}\int_{t_{k}}^{t_{k+1}}\Phi_{13}\left(t_{k+1},\tau \right)d\tau \\ Q_{22} & =\sigma_{v}^{2}\int_{t_{k}}^{t_{k+1}}\Phi_{22}^{2}\left(t_{k+1},\tau \right)d\tau +\sigma_{u}^{2}\int_{t_{k}}^{t_{k+1}}\Phi_{23}^{2}\left(t_{k+1},\tau \right)d\tau \\ Q_{23} & =Q_{32}=\sigma_{u}^{2}\int_{t_{k}}^{t_{k+1}}\Phi_{23}\left(t_{k+1},\tau \right)d\tau \\ Q_{33} & =\sigma_{u}^{2}\int_{t_{k}}^{t_{k+1}}1d\tau . \end{array}$$

Note that the units of $\left\{\sigma_{v},\sigma_{u}\right\}$ must agree with those provided in Table 2 to ensure that these products are dimensionally consistent. Neglecting the contribution of these integrals will result in misrepresentation of the random process parameters. To proceed, the integrals in the above expressions must be evaluated. These have each been evaluated analytically, giving the following:

(A12) $$\begin{array}{cc} \int_{t_{k}}^{t_{k+1}}\Phi_{12}^{2}\left(t_{k+1},\tau \right)d\tau & =\frac{1}{4p_{1}^{2}}\left[\frac{1}{p_{2}}\left(1-e^{-2p_{2}dt}\right)-\frac{1}{\omega^{2}}\left(p_{2}-e^{-2p_{2}dt}\left(p_{2}\cos\left(2p_{1}dt\right)-p_{1}\sin\left(2p_{1}dt\right)\right)\right)\right]\\ \int_{t_{k}}^{t_{k+1}}\Phi_{13}^{2}\left(t_{k+1},\tau \right)d\tau & =\frac{1}{4p_{2}p_{1}^{2}\omega^{10}}[e^{-2p_{2}dt}\left(p_{2}\left(p_{2}^{3}-3p_{1}^{2}p_{2}\right)\cos\left(2p_{1}dt\right)+p_{2}\left(p_{1}^{3}-3p_{1}p_{2}^{2}\right)\sin\left(2p_{1}dt\right)-\omega^{4}\right)\\ \; & +e^{-p_{2}dt}\left(16p_{1}^{2}p_{2}^{2}\cos\left(p_{1}dt\right)+8p_{1}p_{2}\left(p_{1}+p_{2}\right)\left(p_{2}-p_{1}\right)\sin\left(p_{1}dt\right)\right)\\ & +p_{1}^{2}\left(p_{2}^{2}\left(4p_{2}dt-11\right)+p_{1}^{2}\left(1+4p_{2}dt\right)\right)] \end{array}$$

(A13) $$\begin{array}{cc} \int_{t_{k}}^{t_{k+1}}\Phi_{22}^{2}\left(t_{k+1},\tau \right)d\tau & =\frac{1}{4p_{2}}-\frac{1}{4p_{1}^{2}p_{2}}\left(e^{-2p_{2}dt}\left[\omega^{2}-p_{2}^{2}\cos\left(2p_{1}dt\right)-p_{1}p_{2}\sin\left(2p_{1}dt\right)\right]\right)\\ \int_{t_{k}}^{t_{k+1}}\Phi_{23}^{2}\left(t_{k+1},\tau \right)d\tau & =\frac{1}{4p_{1}^{2}}\left[\frac{1}{p_{2}}\left(1-e^{-2p_{2}dt}\right)-\frac{1}{\omega^{2}}\left(p_{2}-e^{-2p_{2}dt}\left(p_{2}\cos\left(2p_{1}dt\right)-p_{1}\sin\left(2p_{1}dt\right)\right)\right)\right] \end{array}$$

(A14) $$\begin{array}{cc} \int_{t_{k}}^{t_{k+1}}\Phi_{12}\left(t_{k+1},\tau \right)\Phi_{22}\left(t_{k+1},\tau \right)d\tau & =\frac{1}{4p_{1}^{2}}e^{-2p_{2}dt}\left[1-\cos(2p_{1}dt)\right]\\ \int_{t_{k}}^{t_{k+1}}\Phi_{13}\left(t_{k+1},\tau \right)\Phi_{23}\left(t_{k+1},\tau \right)d\tau & =\frac{e^{-2p_{2}dt}}{2p_{1}^{2}\omega^{6}}{\left[p_{1}\cos\left(p_{1}dt\right)+p_{2}\sin\left(p_{1}dt\right)-p_{1}e^{p_{2}dt}\right]}^{2} \end{array}$$

(A15) $$\begin{array}{cc} \int_{t_{k}}^{t_{k+1}}\Phi_{13}\left(t_{k+1},\tau \right)d\tau & =\frac{1}{\omega^{4}}\left[\omega^{2}dt-2P_{2}+e^{-P_{2}dt}\left(2P_{2}\cos\left(P_{1}dt\right)+\left(\frac{P_{2}^{2}}{P_{1}}-P_{1}\right)\sin\left(P_{1}dt\right)\right)\right]\\ \int_{t_{k}}^{t_{k+1}}\Phi_{23}\left(t_{k+1},\tau \right)d\tau & =\frac{1}{P_{1}\omega^{2}}\left[P_{1}-e^{-P_{2}dt}\left(P_{2}\sin\left(P_{1}dt\right)+P_{1}\cos\left(P_{1}dt\right)\right)\right] \end{array}$$

where $dt=t_{k+1}-t_{k}$. Alternatively, elegant solutions for the computation of integrals involving the matrix exponential have been developed by Van Loan [42]. Using the system matrix in Equation (4), the covariance can be computed simply as:

(A16) $$\begin{array}{cc} Q & =\int_{0}^{dt}e^{A\tau }Q_{c}e^{A^{T}\tau }d\tau =F_{3}^{T}\left(dt\right)G_{2}\left(dt\right) \end{array}$$

(A17) $$Q_{c}=\left[\begin{array}{ccc} 0 & 0 & 0\\ 0 & \sigma_{v}^{2} & 0\\ 0 & 0 & \sigma_{u}^{2} \end{array}\right]$$

(A18) $$\begin{array}{c} \exp\left(\left[\begin{array}{cc} -A & Q_{c}\\ 0 & A^{T} \end{array}\right]dt\right)=\left[\begin{array}{cc} F_{2}\left(dt\right) & G_{2}\left(dt\right)\\ 0 & F_{3}\left(dt\right) \end{array}\right] \end{array}$$

Using either of these closed form solutions for the covariance matrix of the oscillator state and bias, the stochastic dynamics can be realized using drawings from a zero-mean Gaussian random vector of unit variance:

(A19) $$\begin{array}{cc} X_{k+1} & =\Phi \left(t_{k+1},t_{k}\right)X_{k}+\Gamma \left(t_{k+1},t_{k}\right){\bar{g}}_{k}+\sqrt{Q}N_{3\times 1}\left(0|1\right) \end{array}$$
